# The Role of HDACs as Leukemia Therapy Targets using HDI

**Published:** 2015-10-01

**Authors:** Ahmad Ahmadzadeh, Elahe Khodadi, Mohammad Shahjahani, Jessika Bertacchini, Tina Vosoughi, Najmaldin Saki

**Affiliations:** 1 Health research institute, Research Center of Thalassemia and Hemoglobinopathy, Ahvaz Jundishapur University of Medical Sciences, Ahvaz, Iran; 2 Department of Surgery, Medicine, Dentistry and Morphology, University of Modena and Reggio Emilia, Modena, Italy

**Keywords:** Histone deacetylases, Histone deacetylase inhibitors, leukemia

## Abstract

Histone deacetylases (HDACs) are the enzymes causing deacetylation of histone and non-histone substrates. Histone deacetylase inhibitors (HDIs) are a family of drugs eliminating the effect of HDACs in malignant cells via inhibition of HDACs. Due to extensive effects upon gene expression through interference with fusion genes and transcription factors, HDACs cause proliferation and migration of malignant cells, inhibiting apoptosis in these cells via tumor suppressor genes. Over expression evaluation of HDACs in leukemias may be a new approach for diagnosis of leukemia, which can present new targets for leukemia therapy. HDIs inhibit HDACs, increase acetylation in histones, cause up- or down regulation in some genes and result in differentiation, cell cycle arrest and apoptosis induction in malignant cells via cytotoxic effects. Progress in identification of new HDIs capable of tracking several targets in the cell can result in novel achievements in treatment and increase survival in patients. In this review, we examine the role of HDACs as therapeutic targets in various types of leukemia as well as the role of HDIs in inhibition of HDACs for treatment of these malignancies.

## Introduction

**Figure 1:**
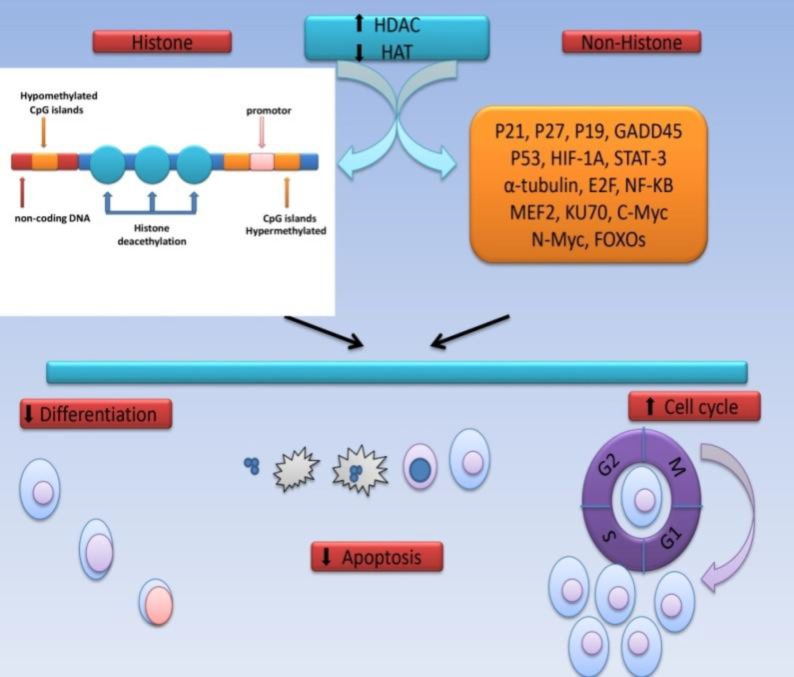
HDAC deacethylation mechanism in leukemic cells

**Figure 2:**
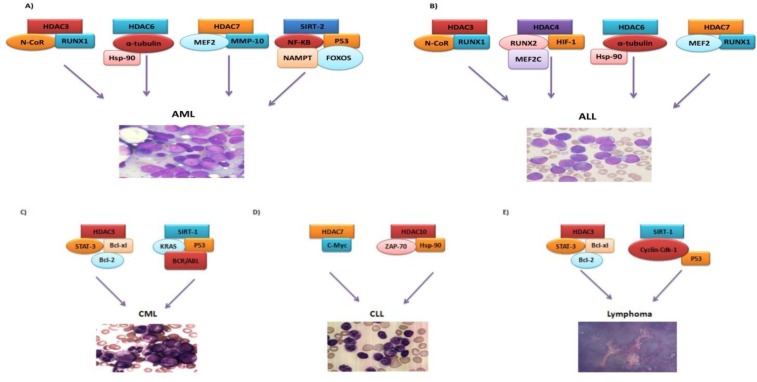
The interaction between HDACs and transcription factors in leukemic cells

 Despite advances in leukemia treatment, leukemias with poor prognosis are refractory to therapy and are associated with relapse. Such cases cause numerous challenges in leukemia therapy.^1^ Genomic analysis in patients has shown that the genes encoding chromatin-modifying proteins form the majority of mutated genes in cancer. Recently, new therapeutic strategies have been established based on epigenetic changes and modification of chromatin structure.^1^^,^^2^ Studies have shown that the development of malignancy is affected by expression of genes as well as changes in DNA sequence. DNA methylation and post-translation modifications in histone, including acetylation, deacetylation and phosphorylation are the most important epigenetic changes in human beings.^3^^,^^4^ Histone acetylation (HAT) and deacetylation (HDAC) are important mechanisms in regulation of gene expression and play a central role in development of human malignancies. Changes in HAT and HDAC enzymes cause disruption of balance between acetylation and deacetylation, which is an important feature in tumor cells.^5^^,^^6^ Hypoacetylation in cancer cells primarily results from increased activity of HDACs than reduced activity of HATs, indicating the prominent role of HDACs in tumor cells (Figure 1).^2^ Chromosomal translocations in leukemias create fusion genes, the protein products of which are involved in development of leukemia via interference with HDACs.^3^^,^^7^^,^^8^ HDACs not only cause deacetylation of histones and transcription of genes but are involved in the regulation of cellular hemostasis, apoptosis, differentiation and cell cycle. Up to now, more than 50 non-histone proteins have been identified as HDAC substrates. These include proteins with regulatory roles in proliferation, migration and cell death (Figure 1).^3^^,^^9^ Human HDACs include 18 enzymes classified in four groups based on structural homology. Class I HDACs include HDAC 1, 3 and 8 which are located in the nucleus. Class II HDACs include IIa (HDAC 4, 7, 6) and IIb (HDAC 6, 10) subgroups, which are located in the nucleus and cytoplasm and are involved in deacetylation of non-histone proteins.^3^^,^^10^ Class III HDACs include SIRT 1-7 (Sirtuins) enzymes, which form a group of proteins with unknown distribution and tissue expression, which can be present in nucleus, cytoplasm and/or mitochondria. Class IV includes the newly detected HDAC 11 (Table 1).^3^^,^^5^^,^^10^ Several studies have indicated the overexpression of HDAC classes in various leukemias, and their overexpression evaluation is currently an important target in identification and treatment of leukemias (Figure 2).^2^^,^^3^^,^^6^

**Figure 3:**
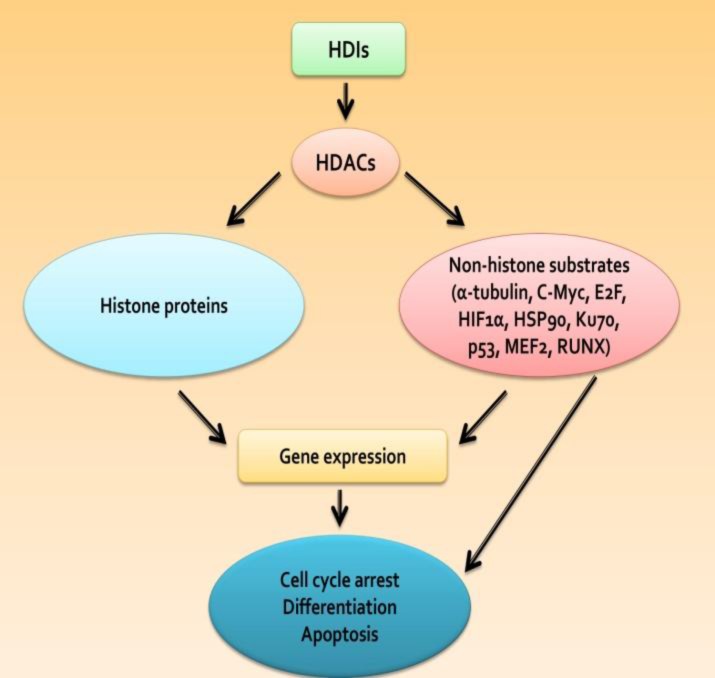
The HDI model of HDAC inhibition in leukemic cells

Histone deacetylase inhibitors (HDIs) are a new class of drugs with a high anti-cancer potential inducing histone acetylation and development of a more open chromatin pattern, which are capable of reactivating tumor suppressor genes. HDIs can also affect the transcription of genes playing a role in cell growth. These inhibitors are classified in different subgroups based on their chemical structure^1^^,^^3^^,^^4^ (Figure 3). In this review, we have discussed HDACs overexpression as therapeutic targets in various types of leukemia as well as use of HDIs in HDACs inhibition for treatment of these malignancies.

**HDACs and their mechanisms of action in leukemia**

The level of homology is the basis for class assignment of HDACs. HDACs deacetylate the lysine residues in histone tails and because a more compact chromatin structure as a result of ionic interactions between positively charged histones and negatively charged DNA, thereby suppressing the gene transcription apparatus.^11^ HDACs can regulate the expression of numerous genes through interference with such transcription factors as E2F, STAT3, P53 and NF-KB. For example, HDAC 1 has been found to inhibit P53 gene function, which results in tumor formation.^3^^,^^12^ The role of Sirtuins (in class III of HDACs) has been recognized in gene expression regulation, apoptosis, response to stress and cell cycle. As the most important member of this family, SIRT1 causes histone deacetylation and transcription regulation of p53 and NFKB genes, resulting in tumor development.^3^^,^^13^ To date, overexpression of a number of HDACs has been detected in prostate, breast and colon cancers. HDACs overexpression causes down regulation of genes involved in regulation of normal proliferation of the cells in a number of hematologic malignancies.^3^^,^^14^^,^^15^ Chromosomal translocations cause oncogenic translation products in various types of leukemia. Studies show that HDACs mediate oncogenic function and inhibit differentiation of hematopoietic cells by multi-protein complexes via affecting gene promoters. Transcription inhibition of tumor suppressor genes like P53 is a usual event in leukemias.^16^

Histone deacetylation enables Histone Methyltransferase (HMT) enzymes to add a methyl group to cytosine in cytosine guanine pairs (CpG), forming CpG islands in DNA. Methylated DNA later forms highly suppressor structures such as heterochromatin (Figure 1).^4^^,^^17^ When the cell progresses towards malignancy, the methylation pattern is totally changed. CpG islands around gene promoters, which are hypermethylated in normal condition of the cell, are hypomethylated and the typically hypomethylated gene promoters are hypermethylated. Therefore, inappropriate promoter methylation and CpG islands leads to tumor suppressor gene silencing and cell transformation (Figure1).^4^^,^^18^ Increased expression of specific HDACs in leukemia results in altered transcription of genes, causing cell proliferation and differentiation in normal conditions (Table 1) (Figure 2).

**HDAC 3**

HDAC 3 belongs to class I HDACs. HDAC 3 is located in the nucleus, is needed for cell growth and is involved in apoptosis process through regulation of pro-apoptotic genes. Therefore, it is of great importance in this group of HDACs.^19^ Overexpression of HDAC 3 in several cancer types has been found to be associated with poor prognosis and response to treatment, including:

**Table 1:** Overexpression of different HDACs in leukemias and targeted-therapy with HDIs

**HDAC**	**Class**	**Localization**	**Leukemia overexpression**	**HDIs**	**References**
**HDAC1**	I	Nucleus	AML, ALL	TSA, SAHA, LAQ-824, PDX-101, LBH-589 ITF2357, FK-228, VPA, Phenyl butyrate, Butyrate**,** AN-9, MS-275, MGCD0103, SK7 041SK7068	(3, 6, 9,10)
**HDAC2**	I	Nucleus	ALL	TSA, SAHA, LAQ-824 PDX-101, LBH-589 ITF2357, FK-228, VPA, Phenyl butyrate, Butyrate, AN-9, MS-275, MGCD0103, SK7068 SK7 041	(3,6,9,10)
**HDAC3**	I	Nucleus	CML, ALL, AML Lymphoma	TSA, SAHA, LAQ-824 PDX-101, LBH-589 ITF2357, FK-228, VPA ,Phenyl butyrate, Butyrate, AN-9, MS-275, MGCD0103, CSO55	(3, 9, 21-23)
**HDAC4**	IIA	Nucleus/Cytoplasm	ALL	TSA, SAHA, LAQ-824 PDX-101, LBH-589 ITF2357, VPA, Phenyl butyrate, Butyrate**, **AN-9	(3, 9, 26)
**HDAC5**	IIA	Nucleus/Cytoplasm	ALL	TSA, SAHA, LAQ-824 PDX-101, LBH-589 ITF2357, Phenyl butyrate, Butyrate**, **AN-9	(3, 6, 9, 26)
**HDAC6**	IIB	Nucleus/Cytoplasm	ALL, AML	TSA, SAHA, LAQ-824 PDX-101, LBH-589 ITF2357**, **AN-9	(3, 9, 30-32)
**HDAC7**	IIA	Nucleus/Cytoplasm	AML, ALL, CLL	TSA, SAHA, LAQ-824 PDX-101, LBH-589 ITF2357, VPA, Phenyl butyrate, Butyrate**, **AN-9	(3, 9, 36-39)
**HDAC8**	I	Nucleus	ALL	TSA, SAHA, LAQ-824 PDX-101, LBH-589 ITF2357, FK-228, VPA, Phenyl butyrate, ButyrateAN-9, MGCD0103	(3, 6, 9, 26)
**HDAC9**	IIA	Nucleus/Cytoplasm	ALL	TSA, SAHA, LAQ-824 PDX-101, LBH-589 ITF2357, VPA, Phenyl butyrate, Butyrate AN-9	(3, 9, 26)
**HDAC10**	IIB	Nucleus/Cytoplasm	CLL	TSA, SAHA, LAQ-824 PDX-101, LBH-589 ITF2357**, **AN-9	(3, 6, 9)
**Sirtuins** **(SIRT1-7)**	III	Nucleus/Cytoplasm Mitochondria	AML, CML, CLL Lymphoma	TV-6, SAHA, FK-228, CSO55	(3, 9, 52)
**HDAC11**	IV	--------------	ALL	------------------------	(3, 9)

**HDAC: **Histone deacetylase; **AML:** Acute Myeloid Leukemia; **ALL:** Acute Lymphoid Leukemia; **CML:** Chronic Myeloid Leukemia; **CLL:** Chronic Lymphoid Leukemia; **HDIs:** Histone deacetylase Inhibitors; **TSA:** Trichostatin A; **SAHA:** suberanilohydroxamic acid; **TV-6:** Tenovin-6

Overexpression of HDAC and decreased expression of HAT in leukemic cells causes deacetylation of histone and non-histone substrates of HDAC. This deacetylation leads to increased cell cycle rate and decreased apoptosis and differentiation in leukemic cells. **HDAC:** Histone Deacetylase; **HDI:** Histone Deacetylase Inhibitor; **GADD45:** Growth Arrest and DNA Damage 45; **HIF -1A:** Hypoxia-inducible factor 1-alpha; **STAT 3:** Signal transducer and activator of transcription 3; **E2F:** Eukaryote transcription 2 Factor; **NF-KB:** nuclear factor kappa-light-chain-enhancer of activated B cells; **MEF2:** myocyte enhancer factor-2; **C-Myc:** C-mycproto-oncogene; **N-Myc:** N-myc proto-oncogene; **OFOXOs:** Forkhead box.

prostate, breast, ovarian and colorectal cancers as well as T-cell acute lymphoid leukemia (T-ALL).^20^^,^^21^ HDAC 3 forms a stable complex with nuclear co-repressor receptor (N-CoR) and enables several biological processes like proliferation, differentiation and apoptosis.^21^ Fusion proteins from chromosomal rearrangements such as t(12; 21) ETV6-RUNX1 in ALL interfere with HDAC3/N-CoR complex, causing increased proliferation of malignant cells (Figure 2.B). Increased expression of HDAC3 in AML is associated with poor prognosis and response to treatment. Certain studies show that chromosomal rearrangements like RUNX1-RUNX1T1 t(8; 21) in AML interfere with N-CoR/HDAC3 and enhance the pathogenesis process in these patients (Figure 2.A).^21^^,^^22^ Increased expression of HDAC3 has also been observed in chronic myeloid leukemia (CML). HDAC3 knockdown causes apoptosis induction in K562 cells in CML. The presence of HDAC3 along with STAT3 in the nucleus, their direct relationship, interference and binding to anti-apoptotic gene promoters like Bcl-xl and Bcl-2 induces the anti-apoptotic activity in leukemic cells (Figure 2.C).^23^^,^^24^

Overexpression of HDAC3 in diffuse large B-cell lymphoma (DLBCL) interferences with STAT3 and causes increased expression of it (Figure 2.E). Inhibition of HDAC3 by HDIs can lead to STAT3 acetylation and phosphorylation inhibition of tyrosine, resulting in inhibition of signal transduction (Table 1).^25^

**HDAC4**

HDAC4 belongs to subclass IIA of HDACs. It is found in the nucleus and cytoplasm, and exerts the majority of its effects by interfering with transcription factors like MEF2C and RUNX2 in the process of skeletogenesis. HDAC4 plays a special role in development of ovarian, breast and kidney tumors. Studies show that silencing of HDAC4 gene reduces the proliferation of tumor cells.^26^^,^^27^ Recently, it has been found that the overexpression of HDAC4 is an important risk factor in T-ALL patients, causing poor response to therapy and resistance to treatment with prednisone. Studies on cells from patients with T-ALL have indicated that HDAC4 knockdown is associated with increased sensitivity to prednisone.^26^^,^^28^ In addition, HDAC4 causes stimulation of HIF-1 (hypoxia-inducible factor-1), enhancing the tumorigenesis process in patients with T-ALL (Table 1) (Figure 2.B).^29^

**HDAC6**

HDAC6 belongs to subclass IIB of HDACs. It is located in the nucleus and cytoplasm, and has histone as well as non-histone substrates such as tubulin or hsp90.^30^^,^^31^^,^^32^ Tubulin deacetylation is essential for many cellular processes, including proliferation and homeostasis. α-tubulin interaction with HDAC6 causes tubulin deacetylation and accumulation of a multi-protein complex containing dynein as microtubule and aggresome.^33^ This complex then transfers the aggresome to lysosomes and causes degradation of the unfolded protein. Tubulin deacetylation also causes accumulation of microtubule complexes, mitotic cell division and proliferation.^31^^,^^32^^,^^33^ Deacetylation of hsp90 by HDAC6 also affects protein degeneration through the aggresome pathway. It affects the function of chaperone on nuclear receptors and causes complex formation, resulting in transcription inhibition.^30^^,^^34^^,^^35^ Overexpression of HDAC6 in AML causes proliferation of malignant cells and resistance of AML cells to cytarabine. Inhibition of HDAC6 by HDIs causes acetylation and degradation of hsp90 chaperone and α-tubulin function in leukemic cells, increasing drug sensitivity of malignant cells to cytarabine (Figure 2.A).^30^^,^^33^ Other studies have indicated the overexpression of HDAC6 in ALL. Inhibition of HDAC6 by HDIs causes α-tubulin acetylation, accumulation of ubiquitinated proteins and subsequent apoptosis of malignant cells in these patients (Figure 2.B) (Table 1).^32^

**HDAC7**

HDAC7 belongs to subclass IIA of HDACs. It is located in the nucleus and cytoplasm. HDAC7 affects the myocyte enhancer factor 2 (MEF2) family of transcription factors, inhibits their transcription and inactivates their target genes like MMP-10, resulting in dysregulation of cell-cell interactions.^36^^,^^37^ Presence of promyelocytic leukemia protein nuclear bodies (PMLNBs) in the nucleus plays a role in regulation of transcription, apoptosis and oncogenesis in AML. Interaction of PMLNBs with HDAC7, which is increased in this type of leukemia, inhibits MEF2 and the function of its target gene of matrix metalloproteinase-10 (MMP-10)^36^^,^^38^ (Figure 2.A). MEF2 family genes are expressed in various stages in differentiation of lymphoid lineage. Research has indicated that overexpression of HDAC7 and its interaction with MEF2 family genes in ALL largely inhibits transcription in lymphoid cells, causing failure of normal differentiation in lymphoid lineage.^39^^,^^40^ Some studies have shown the relationship between overexpression of HDAC7 and t(12; 21) ETV6-RUNX1 in ALL. HDAC7 binds hypoxia-inducible factor 1a (HIF1A), increasing apoptosis and transcription in malignant cells in ALL (Figure 2.B).^39^^,^^41^ Overexpression of HDAC7 is associated with a poor prognosis in chronic lymphoid leukemia (CLL). It binds C-Myc transcription factor gene, causing its overexpression and increased proliferation of malignant cells in CLL (Figure 2.D) (Table 1).^42^^,^^43^

**SIRT-1**

SIRT-1 is a member of Sirtuins (SIRT 1-7) family of class III HDACs, which is located in the nucleus. SIRT-1 is NAD^+ ^dependent and is involved in many cellular processes from DNA repair, cell cycle and metabolism to cancer process, aging and cell survival under stress conditions through deacetylation of histone and non-histone substrates.^41^^,^^44^ In addition, SIRT-1 has been found to play an important role in rare genetic mutations, pathogenesis of solid tumors and leukemias as well as drug resistance. It can regulate the acetylation of genes in several transcription factors, including P53, Ku70, FOXOs, E2F1, NF-KB, C-Myc and N-Myc, which may lead to proliferation of malignant cells.^44^^,^^45^^,^^46^ The precise role of SIRT-1 in malignancies depends on tumor type and P53 involvement. SIRT-1 deacetylates the lysine residues in P53, inhibiting its transcription as well as formation of malignant cells.^44^^,^^47^ Research has indicated that BCR/ABL and overexpression of KRAS oncogene in blastic phase of CML in malignant progenitors lead to overexpression of SIRT-1, which is necessary for BCR/ABL leukemogenesis.^44^^,^^45^^,^^46^ In addition, BCR/ABL causes increased production of reactive oxygen species, double strand DNA breaks and reduced DNA repair in interaction with SIRT-1 and is a factor of drug resistance in malignant progenitors in CML.^45^^,^^48^ Moreover, interaction of BCR/ABL with SIRT-1 leads to P53 deacetylation and results in development of tumor. Inhibition of SIRT-1 by HDIs causes proliferation reduction of malignant progenitors in CML and decreased drug-resistance to Imatinib (Figure 2.C).^44^^,^^49^^,^^50^ Phosphorylation of SIRT-1 during the cell cycle regulates its deacetylase activity. Phosphorylation of SIRT-1 by Cyclin/Cdk1 causes SIRT-1 overexpression in the cell cycle and increases the proliferation of malignant cells in adult T-cell leukemia-lymphoma (ATL), playing a critical role in pathogenesis of this disease.^44^^,^^51^ (Figure 2.E) (Table 1). Other HDACs involved in leukemia have been mentioned in Table 1 and Figure 2. In general, evaluation of the impact of HDACs in development of drug resistance, relapse, prognosis and response to treatment can indicate their important role as therapeutic targets of HDIs in leukemic cells.

**Structure and classification of HDIs**

HDIs form a novel class of anti-tumor drugs in treatment of leukemia. They are cytotoxic agents inducing differentiation, apoptosis and cell cycle arrest in malignant cells by inhibiting HDACs, preventing transformation of normal cells toward tumor cells. HDIs have been developed in an attempt to upregulate the genetically silent genes causing leukemic phenotype.^6^^,^^53^ They are structurally classified in four groups including hydroxamates, cyclic peptides, aliphatic acids and benzamides.^6^^,^^9^ In recent years, HDIs have shown an important anticancer effect in a number of solid tumors and hematologic malignancies. Single agent or combined treatment of HDIs with other anti-tumor agents is a novel treatment strategy for treating children and adults with leukemia.^6^^,^^54^ In hydroxymate group, trichostatin-A (TSA) was the first drug with a recognized effect in inhibition of HDACs. Vorinostat (SAHA) is structurally similar to TSA, and was the first HDI approved for clinical use by food and drug administration. It is a pan-inhibitor for class I and II HDACs.^6^^,^^55^^,^^56^ M- carboxycinnamic acid bishydroxamate is a potent HDI with such derivative inhibitors as LAQ-824, Panobinostat (LBH-589) and PXD-101, which inhibit class I and II HDACs. Givinostat (IT F2357) is a newly recognized inhibitor in this group. It functions 

**A) **Over expression of HDACs 3, 6, 7 and SIRT-2 as well as interaction with related transcription factors observed in AML. **B)** Overexpression of HDACs 3, 4, 3, 7 and interaction with related transcription factors observed in ALL. **C) **Overexpression of HDAC 3 and SIRT-1 and interaction with related transcription factors observed in CML. **D) **Overexpression of HDACs 7, 10 and interaction with related transcription factors in CLL. **E) **Overexpression of HDACs 3 and SIRT-1 and interaction with related transcription factors in lymphoma. **HDAC:** Histone Deacetylase; **N-CoR:** nuclear co repressor receptor; **RUNX1:** Runt-related transcription factor 1; **HSP-90:** Hot Shock Protein-90; **MEF2:** myocyte transcription factors enhancer factor 2; **MMP-10 :** matrix metalloproteinase; **SIRT:** Sirtuin; **NAMPT:** nicotinamide Phosphoribosyl transferase; **NF-KB:** nuclear factor kappa-light-chain-enhancer of activated B cells; **OFOXOs:** Forkhead box; **HIF -1A:** Hypoxia-inducible factor 1-alpha; **MEF2C:** Myocyte-specific enhancer factor 2C; **MEF2:** Myocyte-specific enhancer factor 2; **STAT 3:** Signal transducer and activator of transcription 3; **Bcl-xl:** B-cell lymphoma-extra large; **Bcl-2:** B-cell lymphoma 2; **KRAS:** V-Ki-ras2 Kirsten rat sarcoma viral oncogene homolog; **BCR/ABL:** breakpoint cluster region protein /Abelson murine leukemia viral oncogene homolog 1; **C-Myc:** C-mycproto-oncogene; **ZAP-70:** (Zeta-chain-associated protein kinase 70; **cyclin-cdk1:** cyclin-cyclin dependent kinases 1; **AML: **Acute Myeloid leukemia; **ALL:** Acute Lymphoblastic Leukemia; **CML:** Chronic Myeloid Leukemia; **CLL:** Chronic Lymphoid Leukemia

HDI inhibits HDAC, which leads to deacetylation inhibition in histone and non-histone substrates, changing gene expression and causing cell cycle arrest, differentiation and apoptosis in leukemic cells.

**HDAC:** Histone Deacetylase; **HDI:** Histone Deacetylase Inhibitor; **C-Myc:** C-myc proto-oncogene; **E2F:** Eukaryote transcription 2 Factor; **HIF -1A:** Hypoxia-inducible factor 1-alpha; **HSP-90:** Hot Shock Protein-90; **MEF2:** myocyte enhancer factor-2; **RUNX:** Runt-related transcription factor.

together with LBH-589 in nanomolar concentrations but their target in the cell has not been recognized yet.^9^^,^^57^^,^^58^ Cyclic peptides form a group of HDIs, including depsipeptides like Romidepsin (FK-228), apicidin and cyclic hydroxamic acids with peptide groups. FK-228 is the most important drug in this group, which functions in millimolar concentrations. ^9^^,^^59^ Aliphatic acids form the last group of inhibitors, which include valproic acid (VPA), butyrate and phenylbutyrate as weak inhibitors acting in millimolar concentrations. Pivaloyloxymethyl butyrate (AN-9) and SNOX-275 are newly developed inhibitors in this group.^9^^,^^60^ Benzamides form the last group of HDIs, including Entinostat (MS-275) and Mocetinostat (MGCD0103). MS-275 selectively affects HDAC1 and 3 but has no inhibitory effect on HDAC6 (Table 1).^9^^,^^61^

**Inhibition mechanism of HDIs in leukemia**

HDIs alter a specific ratio of the genes expressed in transformed cells. This effect on gene transcription results from acetylation of a certain complex of histones with other proteins, which regulates gene expression. A number of genes are downregulated by HDIs. P21 cyclin-dependent kinase inhibitor is one of the most common genes induced by HDIs.^9^^,^^62^ Induction of p21 is independent of P53. It occurs through increased histone acetylation in H3K4 in p21 promoter region. The protein complex associated with proximal region in p21e promoter includes HDAC1 and 2, Br g-1, GCN5, BAF155, myc, p300 and SP1. These findings suggest that selective change in gene transcription by HDIs is determined by characteristics of proteins in transcription factor complexes like HDACs.^9^^,^^62^^,^^63^ HDIs cause selective changes in the expression of HDAC class II proteins in addition to catalytic regions of HDACs. For example, HDAC7 is selectively downregulated by at least two different HDIs, including Vorinostat and Depsipeptide. This inhibition is associated with downregulation of HDAC7 mRNA and growth arrest in transformed cells.^9^^,^^64^ Cell death and growth inhibition mechanism of transformed malignant cells by HDIs has not been completely understood. HDIs can generate a set of acetylated histones and non-histone proteins involved in the regulation of cell proliferation, gene expression, cell death and migration. Normal cells are resistant to cell death by HDIs, whereas a wide range of transformed cells are sensitive to cell death through inhibitory effect of HDIs, which can cause cell cycle and differentiation arrest in malignant cells.^65^^,^^66^ HDIs destroy malignant cells by activating intrinsic and extrinsic apoptotic pathways, mitosis arrest, autophagic cell death and ROS induced cell death. HDIs can also cause angiogenesis arrest. Therefore, the special inhibitory effect in transformed cells seems to be dependent upon: (1) cell content (for example, molecular changes) (2) HDI type (3) concentration and (4) contact time with inhibitor (Figure 3).^67^,^68^ Moreover, the ability of HDIs to affect gene expression causes a relaxed structural chromatin by acetylation of promoter regions, downregulating the expression of several genes.^63^^,^^69^ Molecular mechanism of HDAC inhibition by HDI in inducing the anti-tumor effect is due to gene transcription regulation by altered chromosome structure. Gene expression profiling analysis showed that SAHA, TSA and MS-275 upregulate p21, α-tubulin and other genes involved in DNA synthesis, apoptosis and cell cycle. ^70^^,^^71^ Furthermore, HDIs cause downregulation of thymidylate synthetase expression in proteosome subunits, cytokines and other transcription-related proteins. They also regulate intracellular signaling pathways involved in controlling cell survival and differentiation.^72^^,^^73^

Several HDIs are currently under clinical trial for treatment of leukemias and lymphomas (Table 2). However, HDIs such as TSA and Terapoxin B are not use in treatment of patients due to their toxicity in pre-clinical trial.^53^ In addition to HDIs currently used in treatment of malignancies; several new HDIs have been introduced. CSO55 is a novel inhibitor causing cell cycle arrest, leading to cell proliferation inhibition as well as apoptosis induction associated with a high activity of intracellular ROS in K562 cells in CML. This drug is structurally similar to MS-275, exerting its inhibitory effect by downregulating the anti-apoptotic proteins of Bcl-2 family, including Bim, PUMA and Bcl-xl (Table 1, 2).^53^^,^^74^ Spirachostatins A, B is a new drug with a specific inhibitory effect on HDAC in leukemia cell lines. Sp-B induces apoptosis via activation of caspase and Bcl-2 as well as induction of histone acetylation. It reduces the S-Phase activity and causes shift to G0/G1 Phase, leading to cell cycle regulation, P21 

mRNA upregulation and induction, which results in apoptosis.^75^

**Table 2:** clinical trial, toxicity and combination therapy of HDIs used in leukemia

**HDIs**	**Clinical trail**	**Toxicity**	**Combination therapy**	**References**
**SAHA**	Pre-clinical: ALL, AML Phase I: AML, ALL lymphoma Phase II: AML, ALL lymphoma	----------- Anemia, thrombocytopenia fatigue, diarrhea -----------	----------- retinoic acid, Etoposide ,Flavopiridol, Pyroxamide, idarubicin decitabine, idarubicin, cytarabine	(4, 6, 9)
**VPA**	Pre-clinical: ALL, AML, lymphoma Phase I: AML Phase II: AML	----------- ----------- -----------	Cytarabine, anthracycline, idarubicin, 5-azacitidina Decitabine, retinoic acid, 5 -azacitidina -----------	(6, 9)
**MS-275**	Pre-clinical: AML, ALL Phase I: AML , ALL Phase II: ALL	----------- Infections, neurologic toxicity -----------	Fludarabine ----------- -----------	(4, 6, 9)
**AN-9**	Pre-clinical: AML, ALL lymphoma phase II: CML	----------- -----------	Daunomycin, daunorubicin -----------	(6, 9)
**ITF2357**	Pre-clinical: AML phase II: CML	----------- -----------	----------- hydroxyurea	(6, 9)
**FK228**	Pre-clinical: AM Phase I: AML phase II: lymphoma	----------- ----------- myelotoxicity, nausea, vomiting, cardiac dysrhythmias	retinoic acid ----------- -----------	(6, 9)
**MGCD0103**	Pre-clinical: AML ALL Phase I: AMl	----------- -----------	----------- -----------	(6, 9)
**LBH589**	Pre-clinical: ALL Phase I: AML, lymphoma phase II: ALL	----------- Cardiac toxicities, nausea diarrhea, vomiting, hypokalemia, loss of appetite thrombocytopenia -----------	----------- ----------- -----------	(4, 6, 9)
**PXD101**	Phase I: ALL	-----------	Azacitidine	(4)
**NSC706995**	Phase I: ALL	-----------	-----------	(4)
**LAQ-824**	Pre-clinical: ALL	-----------	-----------	(4, 72)
**CSO55**	Pre-clinical: CML Phase I: CML Phase II: lymphoma Phase III: lymphoma	-----------	-----------	(53)
**Spirachostatins A, B**	Pre-clinical: ALL CML, lymphoma	-----------	-----------	(75)
**TSA**	Pre-clinical: ALL CML, lymphoma	Anemia, thrombocytopenia fatigue, diarrhea	-----------	(75)
**Sodium butyrate**	Pre-clinical: ALL, CML			(4, 5, 43)

 New synthetic drugs with demonstrated inhibitory effect on HDAC1, 2 include SK7 041 and SK7068 (Table 1, 2).^9^ All cancer cells have multiple defects in the expression and/or structure of proteins regulating cell proliferation, migration and death. There is also heterogeneity in multiple defects between transformed cells in a malignancy. Therefore, a higher level of target proteins in HDIs can cause better performance against a wide range of hematological malignancies.^9^^,^^76^ HDIs are also used in synergy with other anti-tumor agents. Their use along with radiotherapy, chemotherapy and epigenetic treatments may play an important role in treatment of malignancies.^9^^,^^69^ For example, VPA increases anti-leukemia activity in AML patients via induction of apoptosis in combination with cytarabine. Synergism between these two drugs as well as DNA damage and the inhibitory effect on Bim protein causes anti-leukemia activity^77^ (Table 2).

## Discussion

 Although the role of HDIs in AML and other leukemias is well established, studies indicate several limitations in the use of HDIs in ALL. Most patients evaluated in the studies show relapse and poor prognosis. Therefore, participants in these studies may not accurately represent the majority of ALL cases. In addition, research often involves those over 18 years of age while ALL mostly afflicts children ^4^^,^^78^ However, these restrictions could open the way to create synthetic drugs with stronger effects in treatment of this type of malignancy. Combination of HDI with other anti-cancer drugs has been the focus of attention. As already mentioned, due to existence of several substrates for HDACs in malignant cells, HDIs can affect a number of targets within the cell. Intelligent HDIs to target several ligands in the cell have been investigated as a substitute for multi-drug compounds since the therapeutic effects of single drugs can be easier predicted.^4^^,^^79^ Overexpression evaluation of each HDAC in leukemias can raise them as treatment targets in a strategy to treat malignancy. In addition, the use of currently recognized HDIs, HDIs with higher performance in combination with other drugs targeting several ligands within the cell can significantly contribute to treatment and longer survival in patients.
